# Supplementary material for: Cryo-EM structures of the human band 3 transporter indicate a transport mechanism involving the coupled movement of chloride and bicarbonate ions
Source: PLoS Biol. 2024 Aug 21;22(8):e3002719. doi: 10.1371/journal.pbio.3002719 (PMC11338459; doi:10.1371/journal.pbio.3002719)
Supplement: S1 Table — (PDF) [file pbio.3002719.s001.pdf]

S1 Table. Cryo-EM data collection, processing, and refinement statistics

Data Set	Cl^- OF-IF	Cl^- IF-IF	HCO_3^- OF-IF	HCO_3^- IF-IF	HCO_3^- OF-OF
**Data collection and processing**					
Magnification			81,000		
Voltage (kV)			300		
Electron Microscope			Krios-GIF-K3		
Defocus range (μm)			-1.0 to -2.5		
Total exposure time (s)			4.0		
Energy filter width (eV)			20		
Pixel size (Å)			1.08		
Total dose ($e^-/Å^2$)			40		
Number of frames			38		
Does rate (e^-/phys. pixel/s)					
No. of initial micrographs	17,814			43,385	
No. of initial particles	10,862,923			48,595,748	
No. of final particles	98,977	106,085	21,892	34,842	16,828
Symmetry	C1	C2	C1	C2	C2
Resolution (Å)	2.99	2.97	3.12	2.99	3.16
FSC threshold	0.143	0.143	0.143	0.143	0.134
Resolution range					
**Refinement**					
Model resolution cut-off (Å)	2.99	2.97	3.12	2.99	3.16
Model composition					
No. of Protein residues	961	906	957	917	1022
No. of ligands	7	6	7	8	10
RMSD[a]					
Bond lengths (Å)	0.004	0.003	0.003	0.002	0.003
Bond angles (°)	0.787	0.623	0.535	0.459	0.531
**Validation**					
MolProbity score	1.31	1.46	1.39	1.60	1.44
Clash score	3.52	5.33	7.05	6.97	8.11
**Ramachandran plot (%)**					
Favored (%)	97.06	96.98	97.99	97.46	98.22
Allowed (%)	2.94	3.02	2.01	2.54	1.78
Disallowed (%)	0	0	0	0	0
CC mask	0.80	0.81	0.82	0.79	0.79
CC box	0.56	0.60	0.59	0.54	0.57
CC vol	0.78	0.77	0.77	0.78	0.77

a, root mean square deviation
