# Supplementary material for: Cryo-EM structures of the human band 3 transporter indicate a transport mechanism involving the coupled movement of chloride and bicarbonate ions
Source: PLoS Biol. 2024 Aug 21;22(8):e3002719. doi: 10.1371/journal.pbio.3002719 (PMC11338459; doi:10.1371/journal.pbio.3002719)
Supplement: S2 Table — (PDF) [file pbio.3002719.s002.pdf]

**S2 Table. Interaction energies within 6.5 Å of bound $Cl^-$**

| Interaction Energy (kcal/mol) | Total | van der Waals | Electrostatic |
|---|---|---|---|
| | -20.06703 | 0.94967 | -21.01670 |
| PRO419 | -0.539956 | -0.209103 | -0.330853 |
| PHE423 | -1.266629 | -0.339030 | -0.927599 |
| PHE464 | 1.009160 | -0.102253 | 1.111414 |
| SER465 | -2.155802 | -0.337763 | -1.818039 |
| GLY466 | -0.829126 | -0.112652 | -0.716474 |
| PRO467 | -0.533946 | -0.036257 | -0.497689 |
| ILE528 | -0.035455 | -0.101452 | 0.065997 |
| PHE532 | -0.339762 | -0.399478 | 0.059716 |
| GLU682 | 4.373999 | -0.082533 | 4.456532 |
| THR727 | 1.043097 | -0.407454 | 1.450551 |
| THR728 | -0.372734 | -0.147564 | -0.225170 |
| VAL729 | -1.990065 | -0.406935 | -1.583130 |
| ARG730 | -17.850712 | 3.816746 | -21.667458 |
| PHE792 | -0.110434 | -0.101647 | -0.008787 |
| MET795 | -0.468663 | -0.082953 | -0.385710 |
