# Supplementary figures and images for: Cryo-EM structures of the human band 3 transporter indicate a transport mechanism involving the coupled movement of chloride and bicarbonate ions
Source: PLoS Biol. 2024 Aug 21;22(8):e3002719. doi: 10.1371/journal.pbio.3002719 (PMC11338459; doi:10.1371/journal.pbio.3002719)

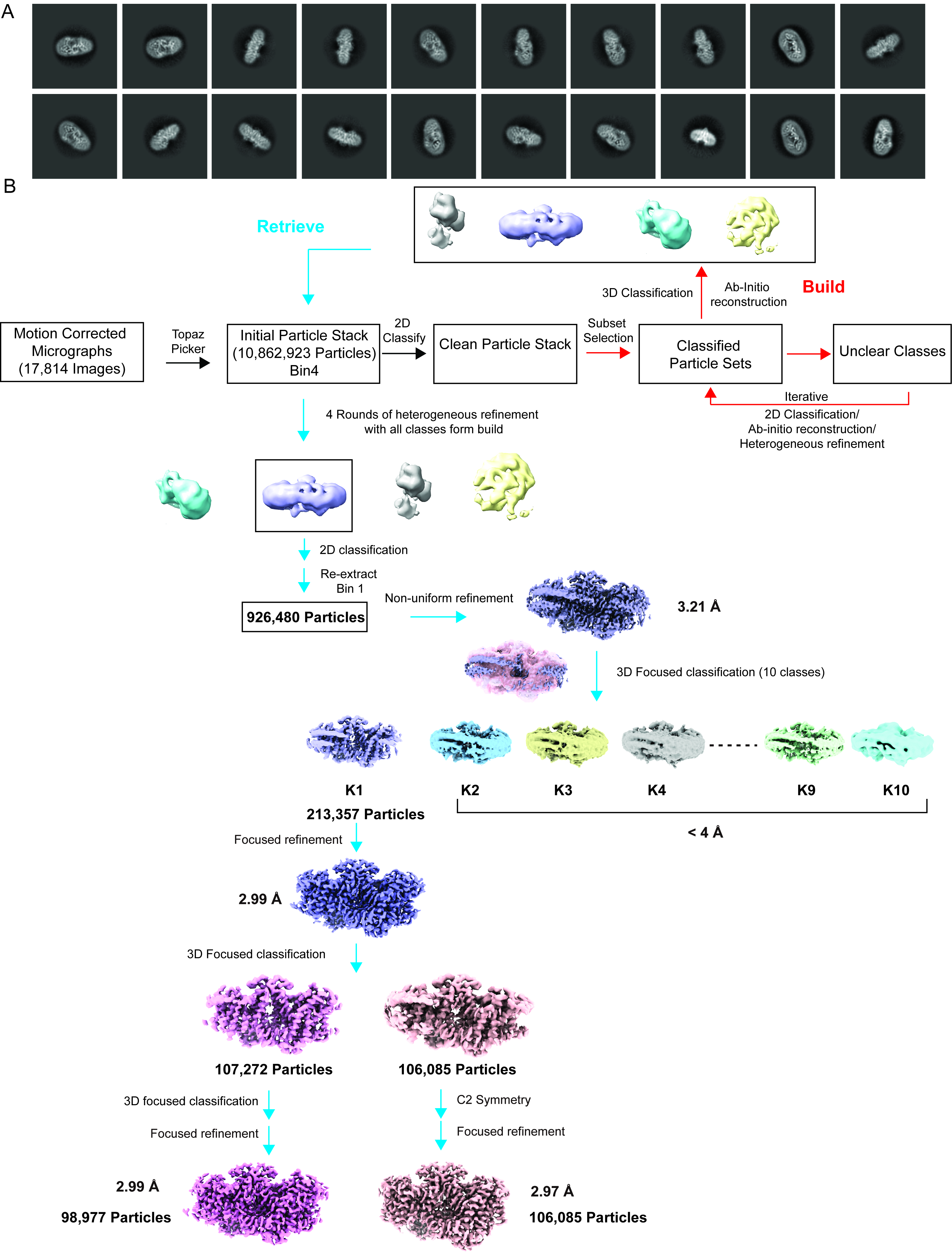

Supplement: S1 Fig — (A) Representative 2D classes of band 3. (B) Processing of 17,814 micrographs using the BaR protocol allowed us to get initial pool of 10,862,923 particles. Further 2D classification led to the selection of 926,480 particles. Nonuniform refinement, 3D focused classification, and focused refinement resulted in the high-resolution structures of band 3 in the OF-IF and IF-IF conformational states. (TIF) [file pbio.3002719.s003.tif]

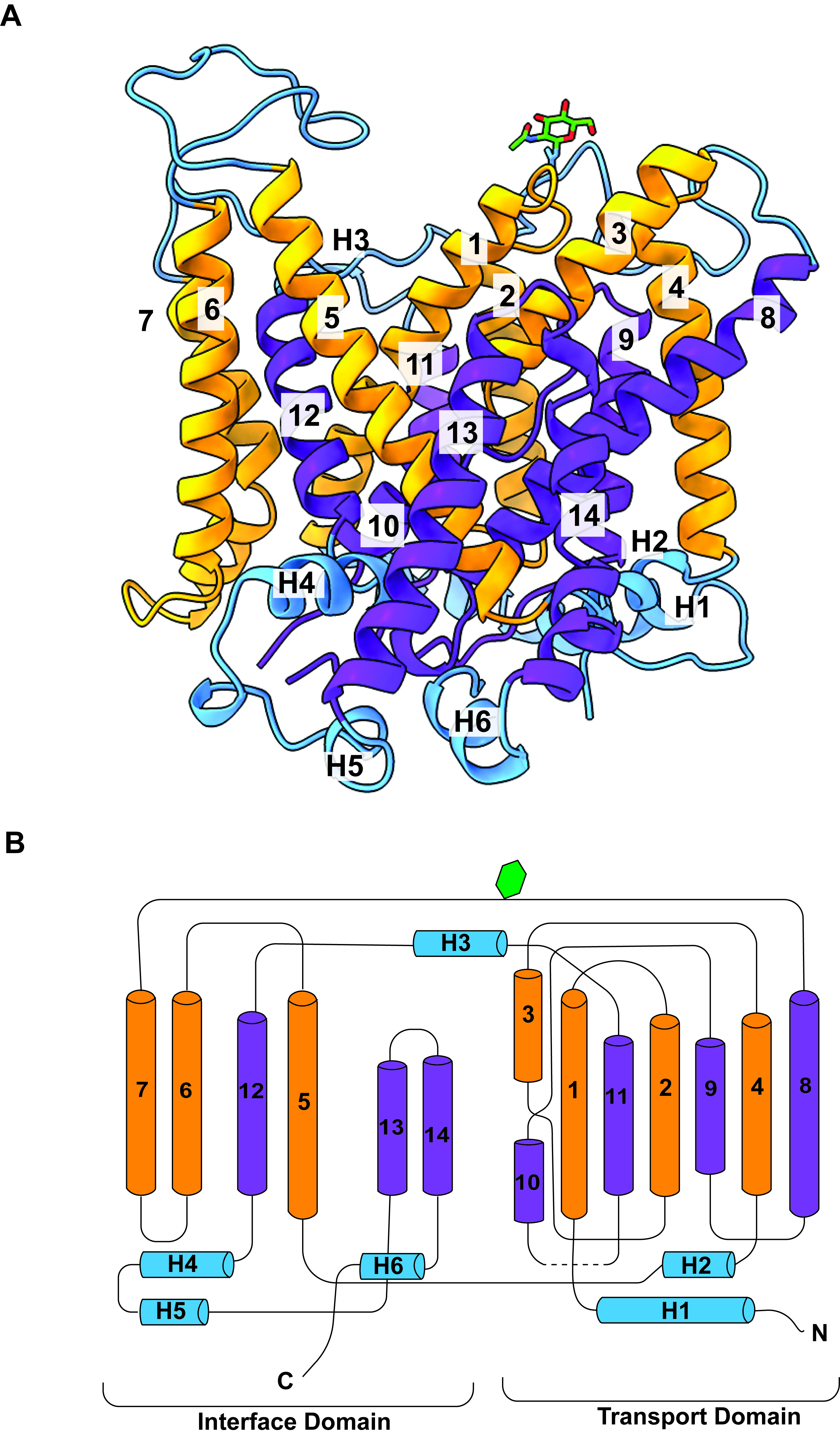

Supplement: S2 Fig — (A) Secondary structural elements of a subunit of band 3. An N-linked glycosylation site is found at residue N642 of each subunit of band 3. (B) Topology of a subunit of band 3. The transmembrane domain of the band 3 subunit contains 14 TMs. The N-linked modification site at residue N642 is highlighted with a green hexagon. In both (A) and (B), TMs 1–7 (orange) and TMs 8–14 (purple) are structurally related to each other in that they are arranged into 2 inverted repeats. (TIF) [file pbio.3002719.s004.tif]

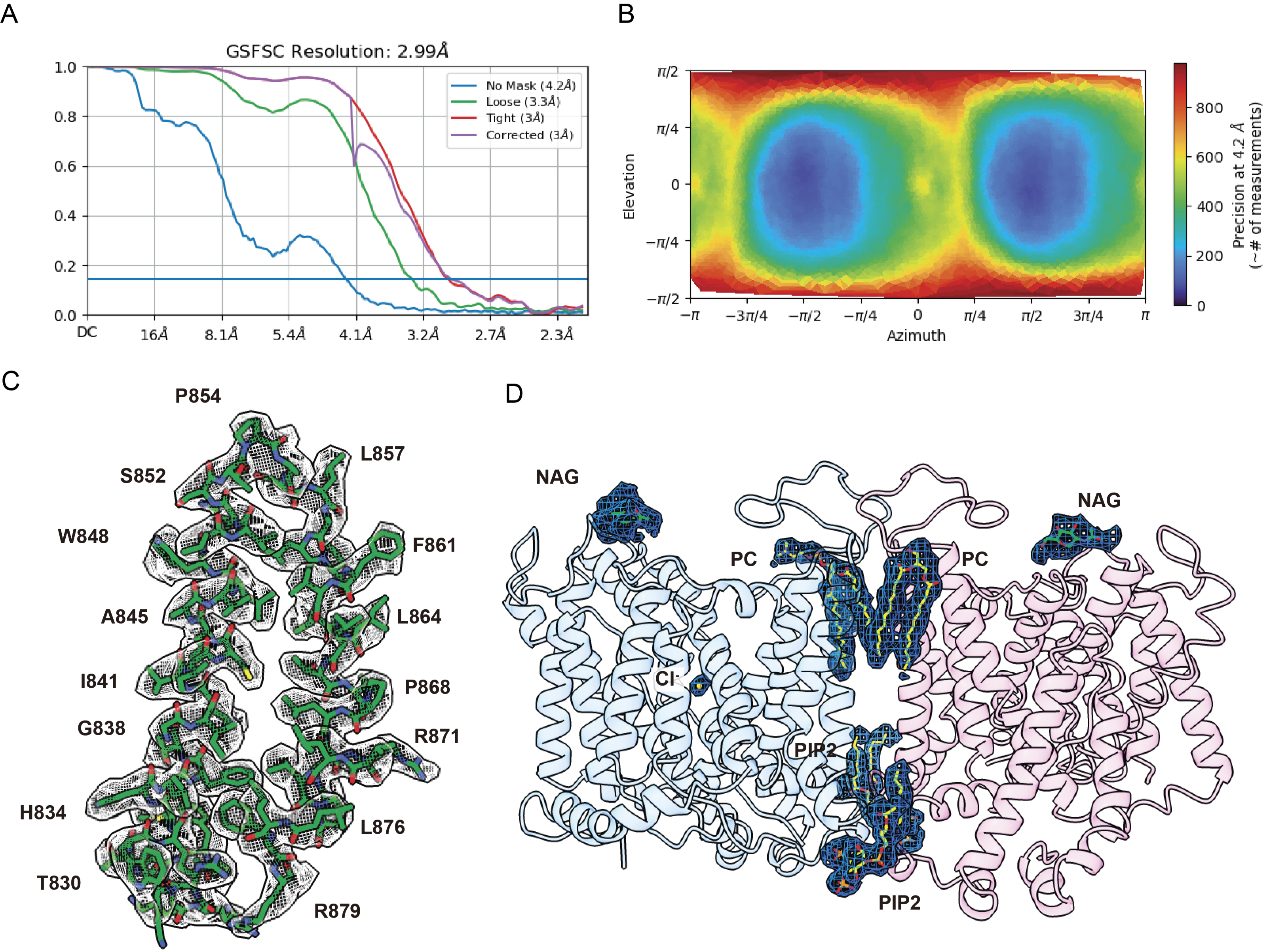

Supplement: S3 Fig — (A) GS-FSC resolution of the cryo-EM map. (B) Euler angle distribution. (C) Local cryo-EM density map of the OF-IF structure of band 3. (D) Cryo-EM density maps of bound Cl-, bound lipids (PC and PIP2), and NAG at the glycosylated modification site. (TIF) [file pbio.3002719.s005.tif]

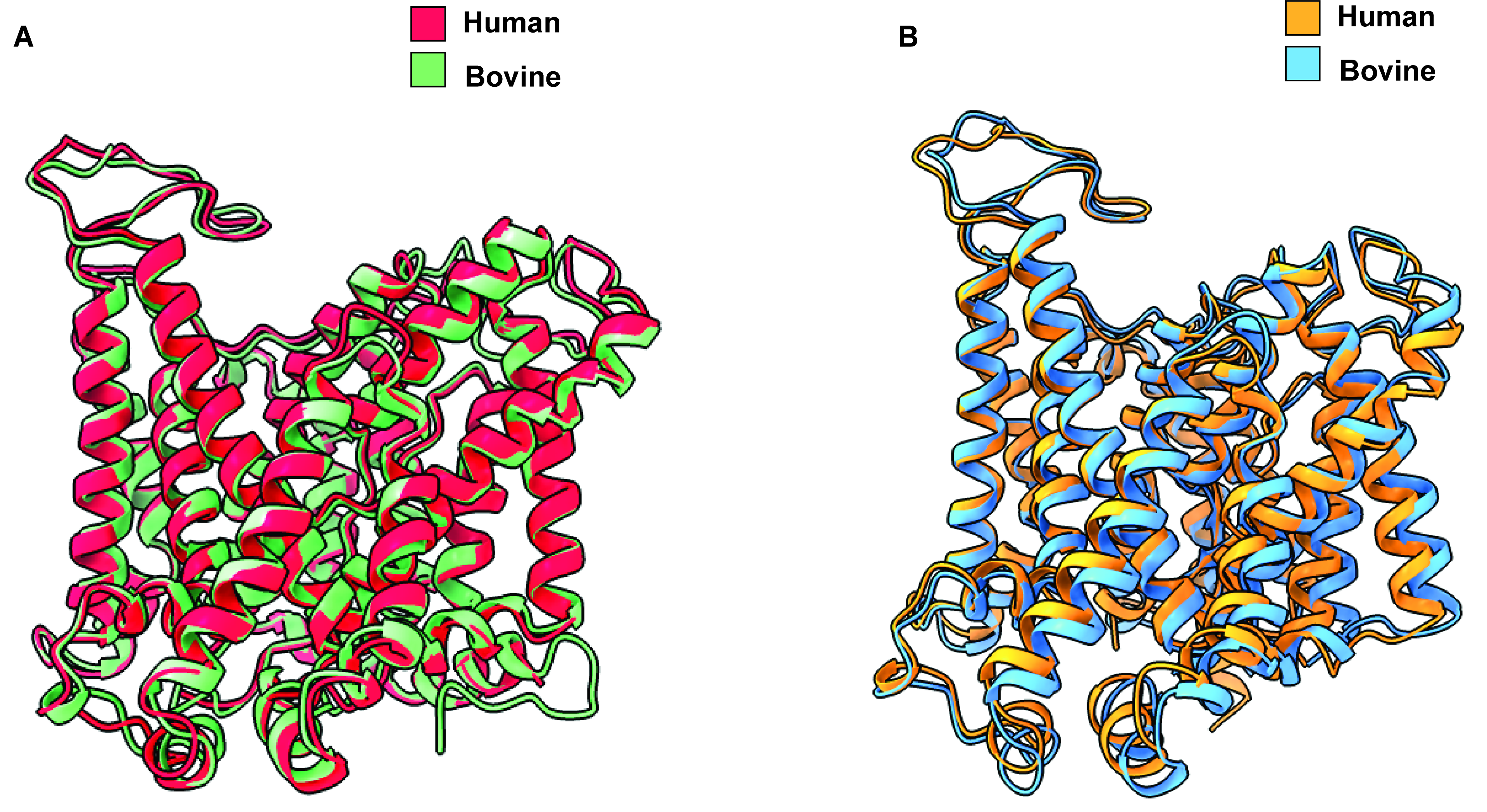

Supplement: S4 Fig — (A) Superimposition of the OF subunit of human band 3 (red) to that of bovine band 3 (green) gives rise to an r.m.s.d. of 1.07 Å (for 422 Cα atoms). (B) Superimposition of the IF subunit of human band 3 (orange) to that of bovine band 3 (cyan) gives rise to an r.m.s.d. of 1.28 Å (for 422 Cα atoms). (TIF) [file pbio.3002719.s006.tif]

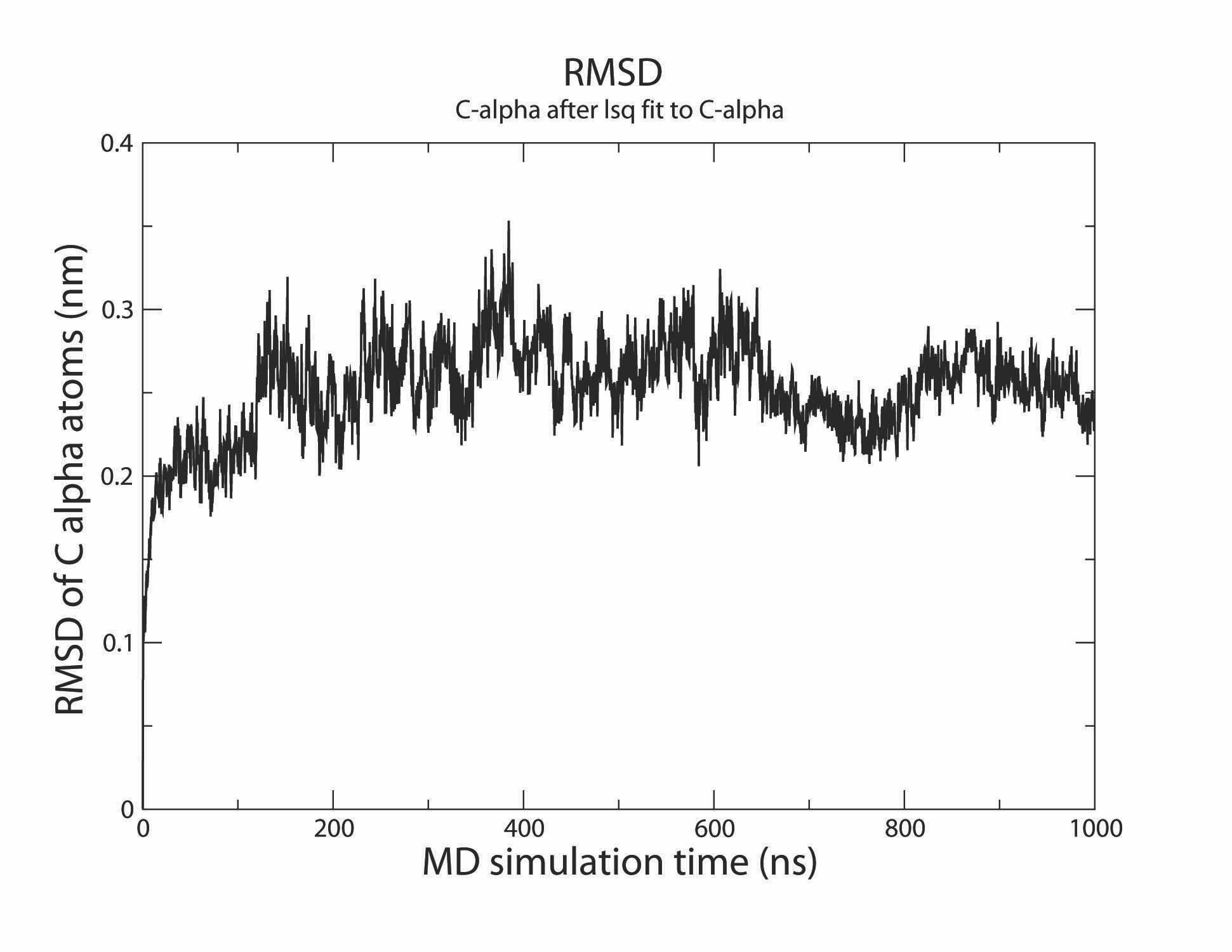

Supplement: S5 Fig — The simulations show results of the OF monomer of band 3 bound with Cl-. The Cα atoms RMSD (root mean square deviation) are based on the MD simulation trajectories (1 μs). The simulations have been done for only one time. (TIF) [file pbio.3002719.s007.tif]

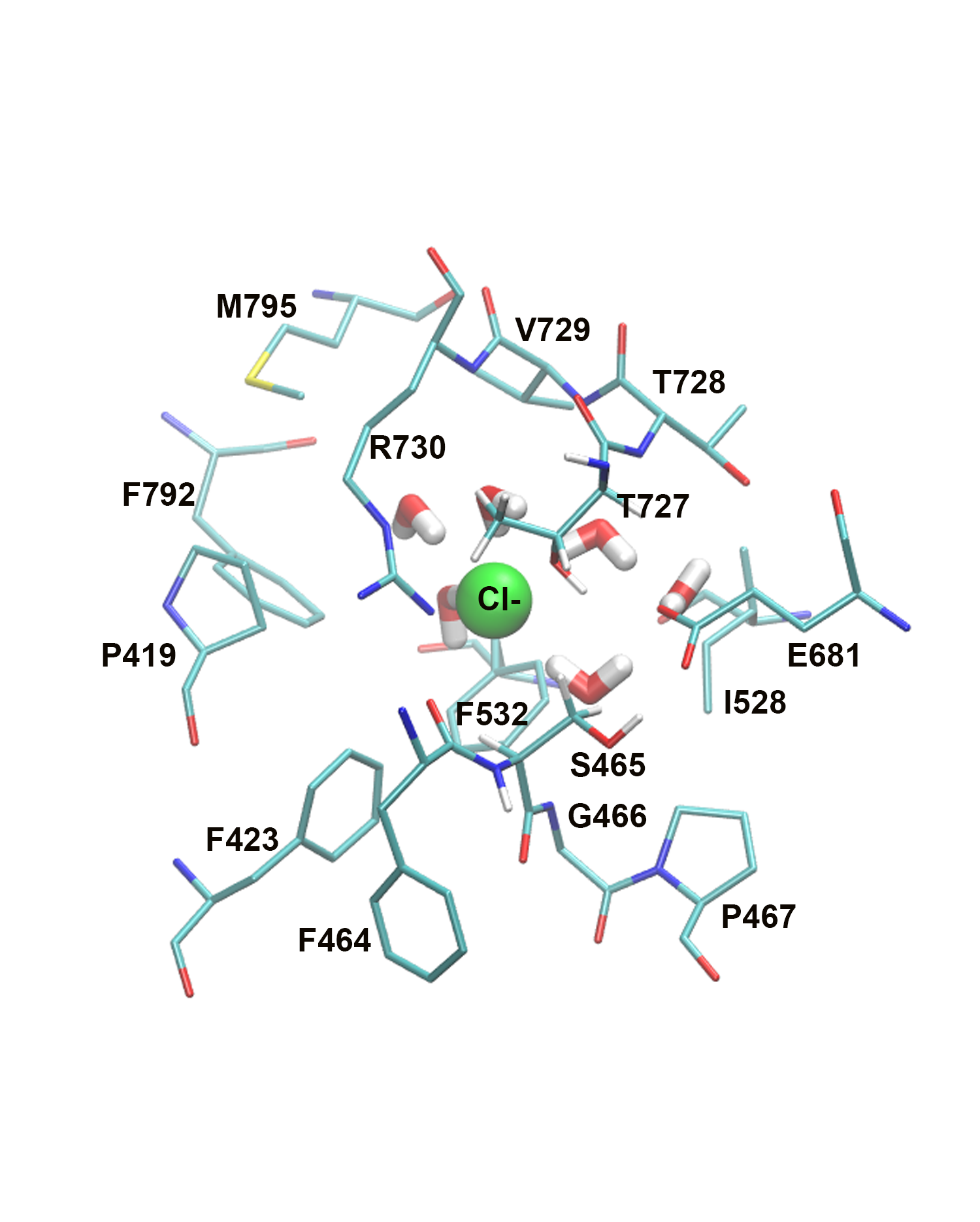

Supplement: S6 Fig — MD simulations suggest that the bound Cl- ion at the Cl- binding site of band 3 is coordinated with 6 water molecules. Important residues responsible for binding Cl- are in cyan sticks. (TIF) [file pbio.3002719.s008.tif]

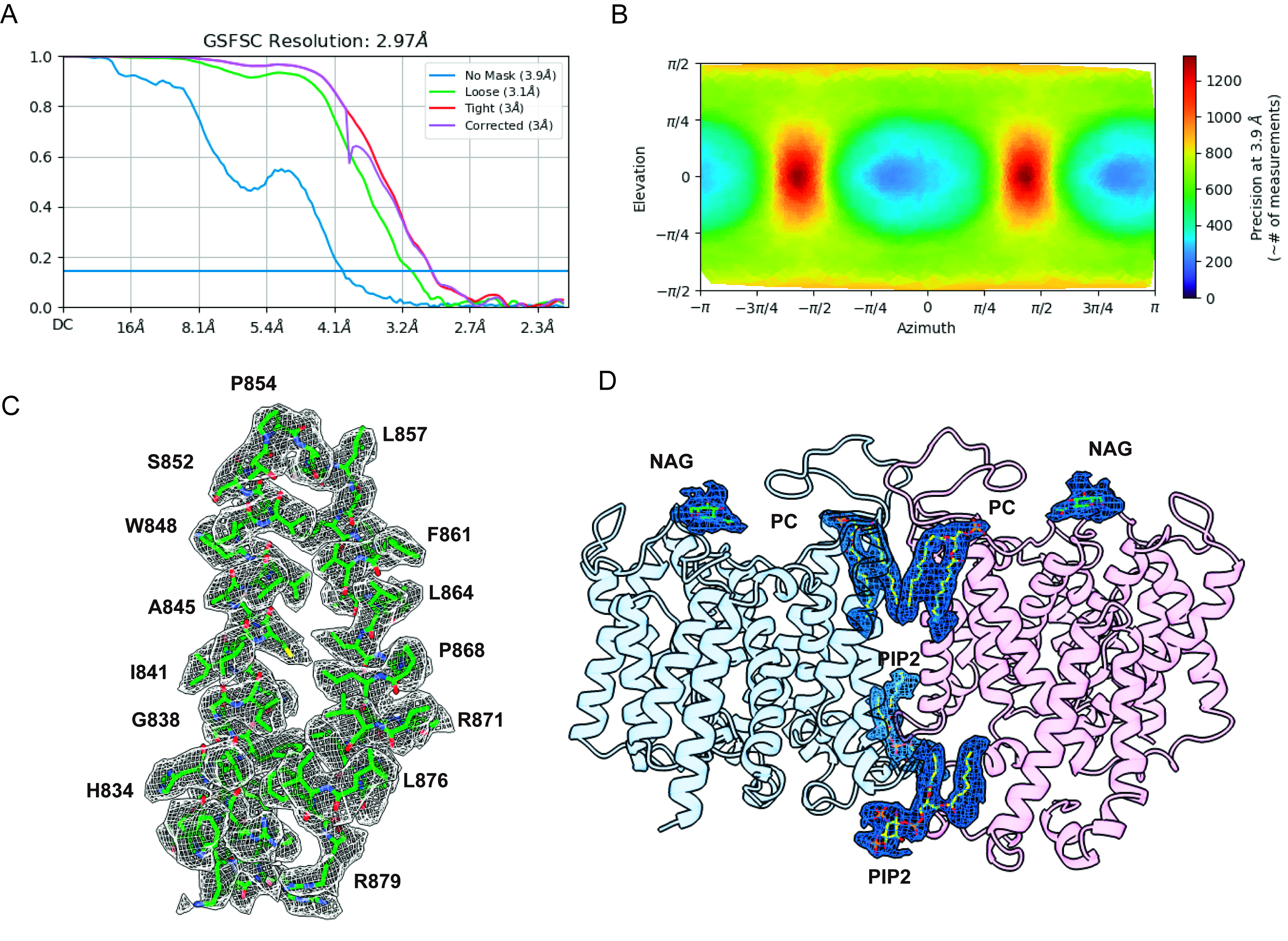

Supplement: S7 Fig — (A) GS-FSC resolution of the cryo-EM map. (B) Euler angle distribution. (C) Local cryo-EM density map of the OF-IF structure of band 3. (D) Cryo-EM density maps of bound lipids (PC and PIP2) and NAG at the glycosylated modification site. (TIF) [file pbio.3002719.s009.tif]

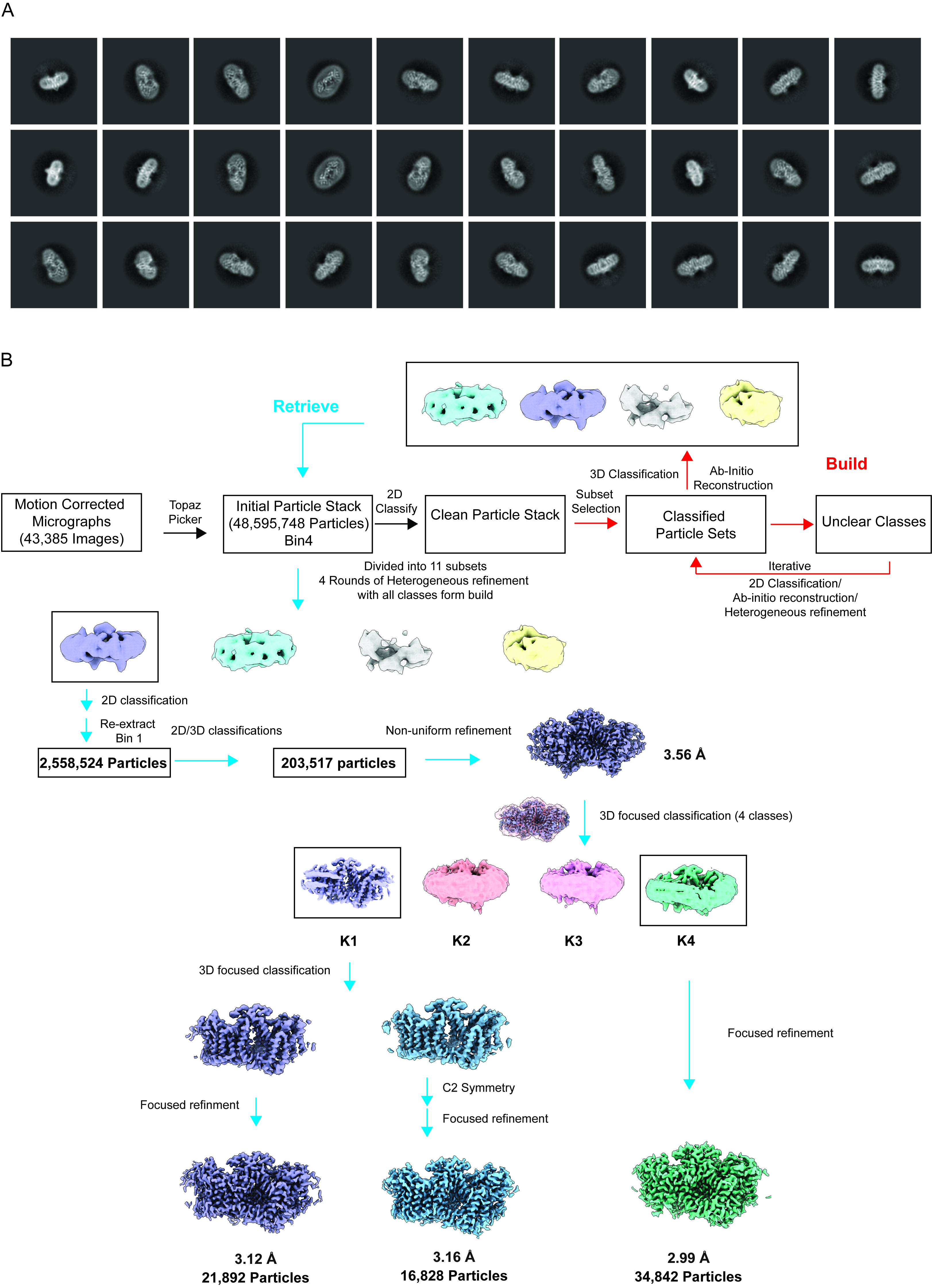

Supplement: S8 Fig — (A) Representative 2D classes of band 3. (B) Processing of 43,385 micrographs using the BaR protocol allowed us to get initial pool of 48,595,748 particles. Further 2D classification led to the selection of 203,517 particles. Nonuniform refinement, 3D focused classification, and focused refinement resulted in the high-resolution structures of band 3 in the OF-IF, IF-IF, and OF-OF conformational states. (TIF) [file pbio.3002719.s010.tif]

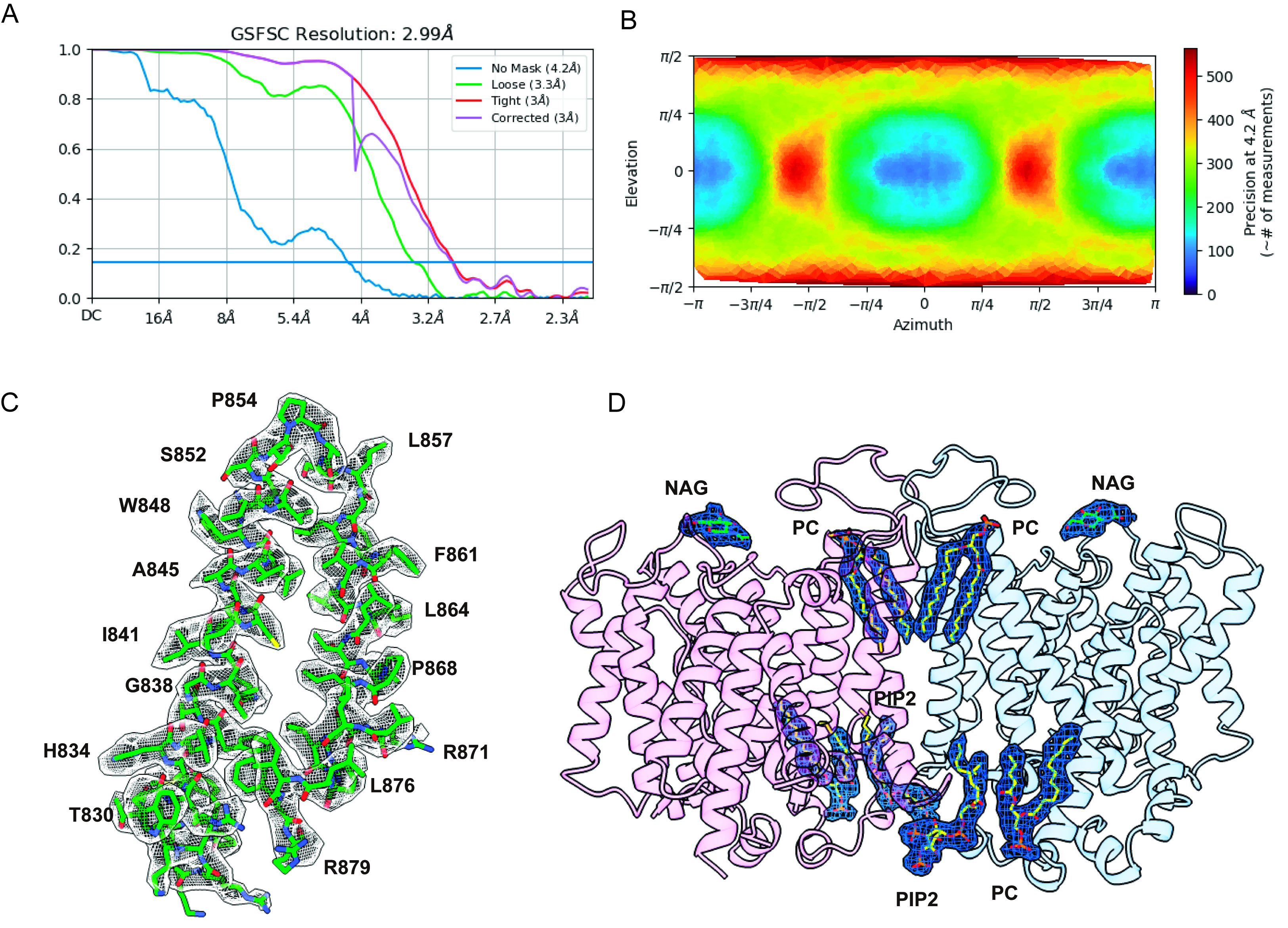

Supplement: S9 Fig — (A) GS-FSC resolution of the cryo-EM map. (B) Euler angle distribution. (C) Local cryo-EM density map of the IF-IF structure of band 3. (D) Cryo-EM density maps of bound lipids (PC and PIP2) and NAG at the glycosylated modification site. (TIF) [file pbio.3002719.s011.tif]

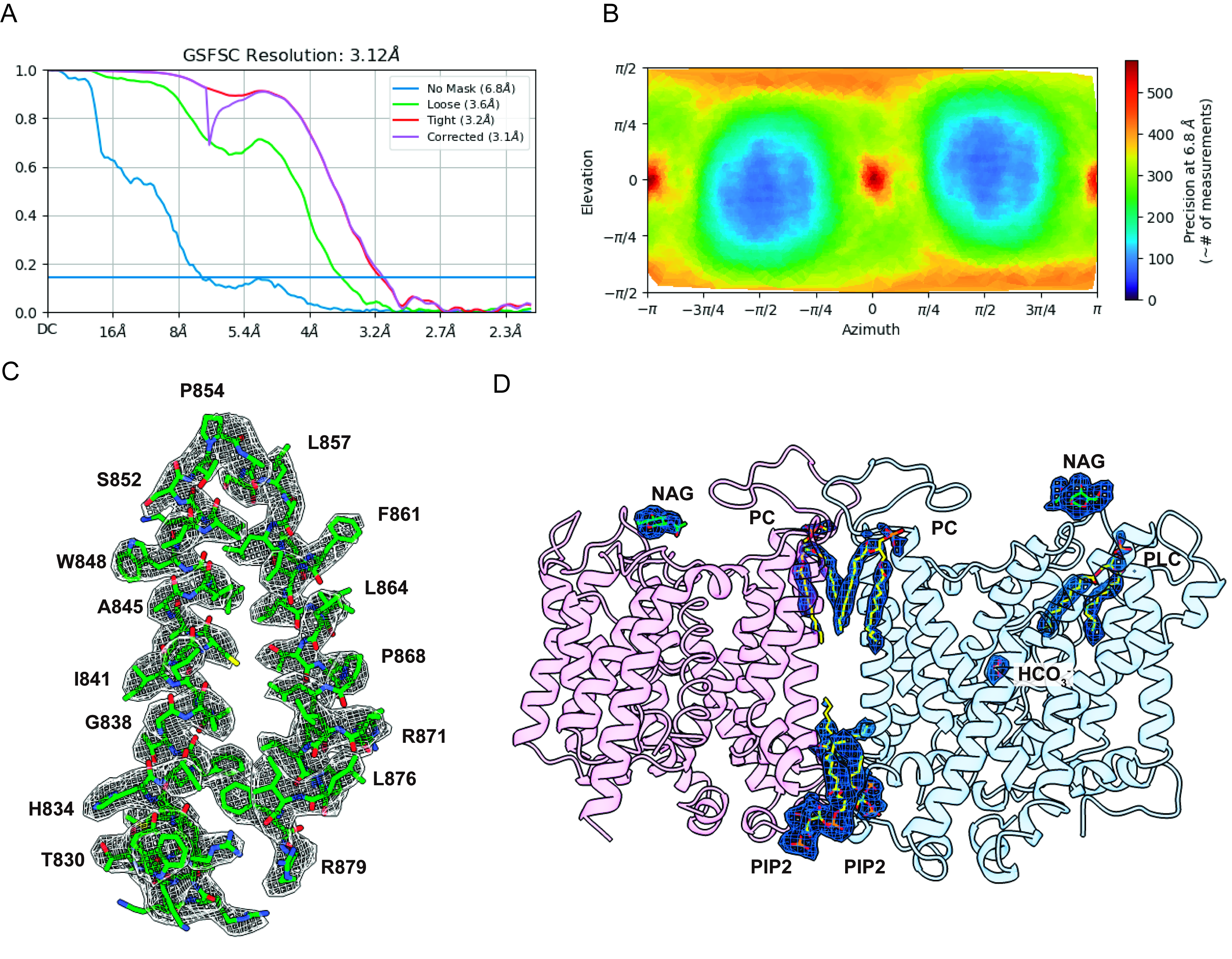

Supplement: S10 Fig — (A) GS-FSC resolution of the cryo-EM map. (B) Euler angle distribution. (C) Local cryo-EM density map of the OF-IF structure of band 3. (D) Cryo-EM density maps of bound HCO3-, bound lipids (PC and PIP2), and NAG at the glycosylated modification site. (TIF) [file pbio.3002719.s012.tif]

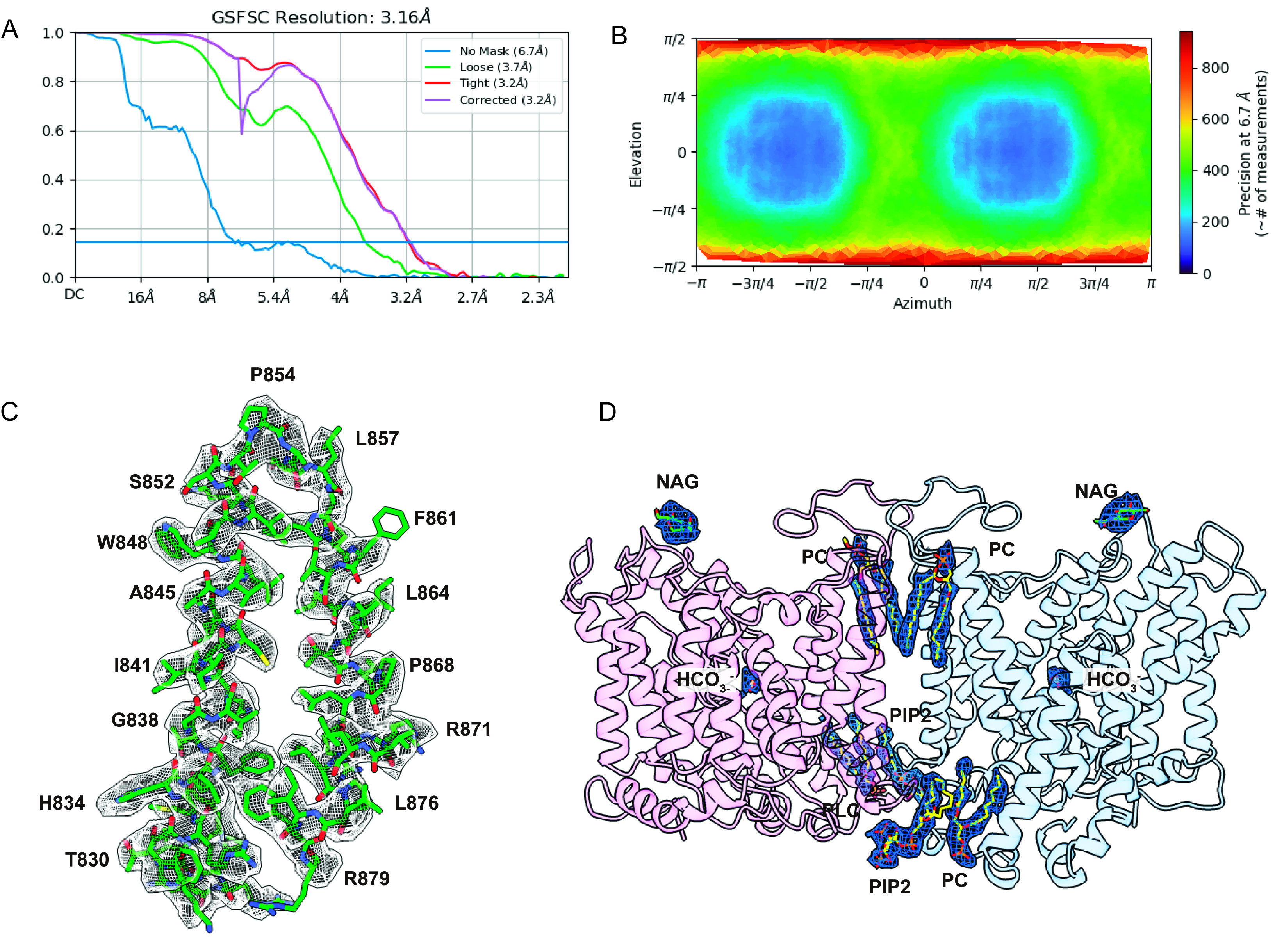

Supplement: S11 Fig — (A) GS-FSC resolution of the cryo-EM map. (B) Euler angle distribution. (C) Local cryo-EM density map of the OF-OF structure of band 3. (D) Cryo-EM density maps of bound HCO3-, bound lipids (PC and PIP2), and NAG at the glycosylated modification site. (TIF) [file pbio.3002719.s013.tif]
