# Supplementary material for: Cryo-EM structures of the human band 3 transporter indicate a transport mechanism involving the coupled movement of chloride and bicarbonate ions
Source: PLoS Biol. 2024 Aug 21;22(8):e3002719. doi: 10.1371/journal.pbio.3002719 (PMC11338459; doi:10.1371/journal.pbio.3002719)
Supplement: Attachment — Submitted filename: Point-by-point response to Academic Editor comments.docx [file pbio.3002719.s016.docx]

Based on our Academic Editor's assessment of your revision, I am pleased to say that we are likely to accept this manuscript for publication, provided you satisfactorily address the remaining comment raised by the Academic Editor regarding the MD simulations (pasted below my signature). Please also make sure to address the following data and other policy-related requests that I have provided below (A-F):

We very much appreciate the Academic Editor’s comment indicating that PLoS Biol is likely to accept our manuscript for publication.

(A) We would like to suggest the following modification to the title:

"Cryo-EM structures of the human band 3 transporter indicate a transport mechanism involving the coupled movement of chloride and bicarbonate ions”

The title of the manuscript has ben changed to "Cryo-EM structures of the human band 3 transporter indicate a transport mechanism involving the coupled movement of chloride and bicarbonate ions”, following the Editor’s suggestion.

(B) You may be aware of the PLOS Data Policy, which requires that all data be made available without restriction: <http://journals.plos.org/plosbiology/s/data-availability>. For more information, please also see this editorial: <http://dx.doi.org/10.1371/journal.pbio.1001797>

All data related to this manuscript are made available to the scientific community without restriction.

Note that we do not require all raw data. Rather, we ask that all individual quantitative observations that underlie the data summarized in the figures and results of your paper be made available in one of the following forms:

-Supplementary files (e.g., excel). Please ensure that all data files are uploaded as 'Supporting Information' and are invariably referred to (in the manuscript, figure legends, and the Description field when uploading your files) using the following format verbatim: S1 Data, S2 Data, etc. Multiple panels of a single or even several figures can be included as multiple sheets in one excel file that is saved using exactly the following convention: S1_Data.xlsx (using an underscore).

-Deposition in a publicly available repository. Please also provide the accession code or a reviewer link so that we may view your data before publication.

All PDB and EMBD codes have been included in this revised manuscript.

Regardless of the method selected, please ensure that you provide the individual numerical values that underlie the summary data displayed in the following figure panels as they are essential for readers to assess your analysis and to reproduce it:

Figure S5

NOTE: the numerical data provided should include all replicates AND the way in which the plotted mean and errors were derived (it should not present only the mean/average values).

As the 1 μs MD simulation is quite expensive and time consuming. We only did this simulation one time. A statement indicating that the simulation has been done only one time has been added in the caption of Figure S5.

(C) Thank you for providing the structural data in the PDB and EMDB database. However, we note that the structures are currently on hold for release. We ask that you please make the structures publicly available at this stage before publication.

We have requested to release all PDB and EMDB files and make them available to the public. These PDBs and EMDBs has been scheduled to be released by May 17th or May 24^th^, 2024.

(D) Please also ensure that each of the relevant figure legends in your manuscript include information on *WHERE THE UNDERLYING DATA CAN BE FOUND*, and ensure your supplemental data file/s has a legend.

Yes, these have been done in this revised version of the manuscript.

(E) Per journal policy, if you have generated any custom code during the curse of this investigation, please make it available without restrictions upon publication. Please ensure that the code is sufficiently well documented and reusable, and that your Data Statement in the Editorial Manager submission system accurately describes where your code can be found.

Please note that we cannot accept sole deposition of code in GitHub, as this could be changed after publication. However, you can archive this version of your publicly available GitHub code to Zenodo. Once you do this, it will generate a DOI number, which you will need to provide in the Data Accessibility Statement (you are welcome to also provide the GitHub access information). See the process for doing this here: <https://docs.github.com/en/repositories/archiving-a-github-repository/referencing-and-citing-content>

There is no custom code generated in this manuscript.

(F) Please ensure that your Data Statement in the submission system accurately describes where your data can be found and is in final format, as it will be published as written there.

Yes, the PDBs and EMDBs have been included, where the readers should be able to find the data of the structures.

------------------------------------------------------------------------

As you address these items, please take this last chance to review your reference list to ensure that it is complete and correct. If you have cited papers that have been retracted, please include the rationale for doing so in the manuscript text, or remove these references and replace them with relevant current references. Any changes to the reference list should be mentioned in the cover letter that accompanies your revised manuscript.

There is no change to the reference list in this revised manuscript.

We expect to receive your revised manuscript within two weeks.

To submit your revision, please go to <https://www.editorialmanager.com/pbiology/> and log in as an Author. Click the link labelled 'Submissions Needing Revision' to find your submission record. Your revised submission must include the following:
-  a cover letter that should detail your responses to any editorial requests, if applicable, and whether changes have been made to the reference list
-  a Response to Reviewers file that provides a detailed response to the reviewers' comments (if applicable, if not applicable please do not delete your existing 'Response to Reviewers' file.)
-  a track-changes file indicating any changes that you have made to the manuscript.

NOTE: If Supporting Information files are included with your article, note that these are not copyedited and will be published as they are submitted. Please ensure that these files are legible and of high quality (at least 300 dpi) in an easily accessible file format. For this reason, please be aware that any references listed in an SI file will not be indexed. For more information, see our Supporting Information guidelines:
<https://journals.plos.org/plosbiology/s/supporting-information> 

*Published Peer Review History*
Please note that you may have the opportunity to make the peer review history publicly available. The record will include editor decision letters (with reviews) and your responses to reviewer comments. If eligible, we will contact you to opt in or out. Please see here for more details:
<https://blogs.plos.org/plos/2019/05/plos-journals-now-open-for-published-peer-review/>

*Press*
Should you, your institution's press office or the journal office choose to press release your paper, please ensure you have opted out of Early Article Posting on the submission form. We ask that you notify us as soon as possible if you or your institution is planning to press release the article.

*Protocols deposition*
To enhance the reproducibility of your results, we recommend that if applicable you deposit your laboratory protocols in [protocols.io](http://protocols.io), where a protocol can be assigned its own identifier (DOI) such that it can be cited independently in the future. Additionally, PLOS ONE offers an option for publishing peer-reviewed Lab Protocol articles, which describe protocols hosted on [protocols.io](http://protocols.io). Read more information on sharing protocols at <https://plos.org/protocols?utm_medium=editorial-email&utm_source=authorletters&utm_campaign=protocols>


Please do not hesitate to contact me should you have any questions.

Kind regards,

Richard

Richard Hodge, PhD
Senior Editor, PLOS Biology
[rhodge@plos.org](mailto:rhodge@plos.org)


------------------------------------------------------------------------

COMMENTS FROM THE ACADEMIC EDITOR

The only remark I have concerns the stated binding affinity of -20 kcal/mol obtained from MD simulations, which would correspond to a Kd of 2 10-15 M which is not realistic. The authors should thus not take this value quantitatively but emphasize that it reflects the general properties of the site to coordinate an anion.

We really appreciate the Academic Editor’s comment. We would like to thank the Editor for catching this mistake. We are very sorry about mistakenly indicating that this is the binding affinity. The -20 kcal/mol is not the binding affinity for Cl^-^ ion, but rather it is the overall interaction energy contributed from surrounding binding residues. The interaction energies were calculated using the Discovery Studio program, in particular using the interaction energy module, which accounts for van der Waals and electrostatic interactions. Additional detailed descriptions of these energy calculations have been added to the method section of the revised manuscript. A new S2 Table was also added to clarify the interactions between bound Cl^-^ and the surrounding band 3 residues.
