# Supplementary material for: Cryo-EM structures of the human band 3 transporter indicate a transport mechanism involving the coupled movement of chloride and bicarbonate ions
Source: PLoS Biol. 2024 Aug 21;22(8):e3002719. doi: 10.1371/journal.pbio.3002719 (PMC11338459; doi:10.1371/journal.pbio.3002719)
Supplement: Attachment — Submitted filename: Point-by-point response to reviewer comments PLoS Biol.docx [file pbio.3002719.s015.docx]

We are submitting a revised version of our manuscript entitled “Transport mechanism of human band 3 involves the obligatorily coupled movement of chloride and bicarbonate.” Four of the referees have raised several important points that we have now addressed in this revision of the paper.

Our revised paper has an expanded scope. An important set of computational MD simulations (1000 ns) has allowed us to validate the chloride binding site observed from our cryo-EM structure. According to the result of the simulations, Cl^-^ is bound stably in this band 3 ion binding site with a strong binding affinity of -20.1 kcal/mol. The simulations also suggest that the Cl^-^ ion is coordinated with six water molecules to further stabilize the binding.

Our point-by-point response to the referees’ comments follows this letter.

The changes are highlighted with blue color in the markup revised manuscript.

We believe that our manuscript has great novelty and provides new mechanistic insight into the function of the band 3 transporter. We also believe that this work is highly significant and will make an impact to the field.

Sincerely,

REVIEWS:

Reviewer #1 (Motoyuki Hattori, signs review): Su et al. reported the cryo-EM structures of the band 3 transporter in multiple conformations, including a newly determined inward conformation. It is noteworthy that they used a self-developed technique called BaR. The quality and significance of the work would merit publication in PLOS biology. Therefore, I recommend this paper for publication if the following concerns are adequately addressed.

We very much appreciate this reviewer’s comment indicating that the “quality and significance of the work would merit publication in PLOS biology”.

Main concerns
1. TM helix numbering labels
Page 6 " TMs 1-7 and TMs 8-14 are structurally related to each other in that they are arranged into two inverted repeats.".
As shown above as an example, the authors have extensively mentioned the numbering of the TM helices, but most of the figures lack this information, so it was often difficult to understand. I strongly recommend the authors to add another figure to show the monomeric structure with the labels for each TM helix together with the membrane topology cartoon. In addition, I would add more labels for the TM helix numbering in Figures 2-6.

In this revised manuscript, a new figure (Fig. 2) showing the topology and structure of a monomer of band 3 has been included. The TM helix numbers have been added in Figs. 2-6.

2. Movies to illustrate the structural changes in the transport cycle

The authors should prepare a movie illustrating the structural changes in the transport cycle using their structures.

A new movie (Movie S1) showing the transition of band 3 from outward facing to inward facing conformations has been added.

3. Page 12. " Surprisingly, we only observed these bound ions in the outward-facing conformation, and
not in the inward-facing state, of the band 3 protomers."

Please explain in detail why the authors did not see bound ions in the inward-facing state.

In this revised version of the manuscript, a few statements “Because of the closing of the internal cavity and dissembling of ion binding sites at the inward-facing conformational state, the binding of these ions at this state may be more transient and lower in affinity. Based on the structural information, the ions are possibly more accessible to solvent at this state. Therefore, it may be difficult to capture the bound Cl^-^ or HCO_3_^-^ at the inward-facing state” have been added in the “Discussion” section (p. 14) to discuss the possibility as to why we did not see bound ions in the inward-facing state.

Reviewer #2: In this contribution by Su et al., the collection of five cryo-EM structures of human band 3 transporter is reported. Although I have no concerns about the quality of generated models, and appreciate the fact that the authors managed to catch this transporter in different conformational states, which certainly improves our understanding of the transport mechanism, I do have some concerns regarding the fine interpretation of their results. This is especially valid for the assignment of cofactors, which  I am very doubtful about. I fail to grasp how the negatively charged side chain of glutamate E681 can be a main binder of a negatively charged chloride ion. The latter should rather repel than being attracted to E681. The same problem is with  the proposed role of E535 as a recruiter of a chloride ion. There is a general problem with cryo-EM studies that there is no direct way to confirm the identity of a ligand, especially when they are small in size and at
relatively low resolution, in contrast to e.g. crystallography, where anomalous dispersion can be measured to confirm the identity. In this particular work it is impossible to unambiguously assign the ligands (especially chloride, to a lesser extent bicarbonate), so the authors should either conduct additional experiments, e.g., MD simulations, transport assays, etc, or they should significantly strip down the discussion of ligand assignment.

We appreciate this reviewer’s comment. However, it is not uncommon to find that a Cl^-^ binding site contains an acidic glutamate or aspartate residue. A census of halide binding sites in protein structures indicates that as many as 30% of these halide binding sites contain an acidic glutamate or aspartate residue (Skitchenko et al. 2020. Bioinformatics 36:3064-3071).

Nonetheless, we added new computational MD simulations data in this revised version of the manuscript. MD simulations indicate that our Cl^-^ binding site is very stable with a strong binding affinity of -20 kcal/mol. In addition to the band 3 amino acid residues, the simulations suggest that the bound Cl^-^ is coordinated with six water molecules which further stabilize the binding.

In addition to the added computational calculation section, the description of the Cl^-^ binding site in this revised manuscript has been extensively modified. Additionally, two new paragraphs have been added on p. 9-10 of this revised version of the manuscript. These two new paragraphs are as follows:

“It is a little surprising that our cryo-EM structure indicates that Cl^-^ ion is anchored by an acidic glutamate residue. However, this is actually not uncommon to find the involvement of negatively charged aspartate or glutamate residue to participate in Cl^-^ binding. For example, a model of the Cl^-^ binding site in the *Aedes aegypti* AAT1 transporter is composed of an aspartate residue (Forrest et al. 2007. PNAS 104:12761-12766). Indeed, it has been observed that there are numerous halide ion binding sites, including those for Cl^-^, Br^-^ and I^-^, each of which contains a negatively charged aspartate or glutamate residue that is actively engaged in halide binding (Skitchenko et al. 2020. Bioinformatics 36:3064-3071). It is also interesting to note that the ion conductance pathway of CLC channels, such as ClC-1, contain a glutamate-tyrosine site in the midway of the channel. It has been proposed that chloride conductance may involve the shuttling of Cl^-^ to the glutamate-tyrosine site (Wang et al. 2019. PLoS Biol 17:e3000218).”

“To validate this chloride binding site in the band 3 exchanger, we performed 1000 ns molecular dynamics (MD) simulations based on the Cl^-^ binding site of our cryo-EM structure. In agreement with our Cl^-^ bound band 3 structure, MD simulations indeed indicate that Cl^-^ is anchored stably in this ion binding site with a strong binding affinity of -20.1 kcal/mol. Our MD simulation also suggests that this bound Cl^-^ ion is coordinated with six water molecules to further stabilize the binding.”

In our cryo-EM structure, the bound chloride ion is actually anchored by at least 9 amino acids to stabilize the binding. A new sentence “The binding of this ion is anchored by at least eight residues, including F423, F464, P419, S465, P467, E681, V729 and R730, which provide hydrophobic and electrostatic interactions to secure Cl^-^ binding” has been added in this revised manuscript (p. 9) to highlight the extensive interactions with different amino acids in the Cl^-^ binding site.

For the sentences in relation to E535, we just wanted to highlight an interesting observation that E681, bound Cl^-^ and E535 line in a straight line. Nonetheless these sentences “Interestingly, in view of the structure of subunit A with the OF conformational state, the side chain oxygen of E535 is 9.7 Å away from bound Cl^-^, where E535, the bound Cl^-^ ion and E681 seemingly assemble as a straight line. There is a chance that E535 helps recruit Cl^-^ from the external environment and then directly transfers this ion to E681 for Cl^-^ binding” have been removed from the text of this revised manuscript.

Minor concerns: please avoid statements of high resolution, 3 Å is anyway not high (even for EM these days)

The word “high” resolution has been removed from this revised manuscript.

In the methods: a typo in the word 'pooled'

This typo has been fixed.

Fig S2/S3/S5 panel C , the colour of the protein or density should be changed

The color of the density has been made much lighter in panel C of supplementary figures Fig. S3, Fig. S7, Fig. S9, Fig. S10 and Fig. S11 oof this revised manuscript (corresponding to Figs. S2, S3, S5, S6 and S7 of the original manuscript). In addition, we have thickened the green colored sticks, which represent the protein amino acid residues, in panel C of these supplementary figures.


Reviewer #3: The manuscript of Su and Yu et al titled "Transport mechanism of band 3 involves the concerted movement of chloride and bicarbonate" reports cryoEM structures of the band 3 protein extracted from human red blood cells and reconstituted into nanodiscs. The purification process seems to produce predominantly homodimeric the human band 3 protein that does not contain any associated proteins. The authors developed and applied a procedure that they call the BaR methodology to tease out multiple conformations within the same data set. In the presence of chloride ion, two conformations were resolved, termed OF-OF and OF-IF, while in the presence of bicarbonate ion, three conformations were resolved, termed OF-OF, IF-IF, and OF-IF. The authors then defined a binding site for chloride and bicarbonate ions, respectively, which relies mainly on the presence or absence of a density at the "internal cavity" where TM3 and TM10 meet. Chloride and bicarbonate ions seem to
occupy discrete sites within the "internal cavity" region, and the position of the bicarbonate ion may shift slightly depending on whether the structure is in IF or OF.

Overall, the work on structure determination is of good quality and the analysis and interpretation of the structures are cautious and reasonable. The work is highly significant for its novel information on mechanisms of anion recognition and exchange, and for the methods developed on isolation of native protein and sorting of particles and building of structural models. Although I would love to see targeted functional validations of novel conclusions on discrete positions of the two anions and the movement of the domains, I understand that these studies are challenging and would take time, and that these could be completed in the future.

We really appreciate this reviewer’s comments, indicating that “the work on structure determination is of good quality” and “the work is highly significant for its novel information on mechanisms of anion recognition and exchange, and for the methods developed on isolation of native protein and sorting of particles and building of structural models.”

I have a few minor comments for the authors to consider:

The name "gate" and "core" domains were given in the initial UraA paper when the mechanism of transport was vague. I think the field of UraA and its structurally homologous relatives has settled on "interface" and "transport" domains.

The words “gate” and “core” have been replaced by “interface” and “transport”, respectively, following this reviewer’s suggestion.

The "internal cavity" between TM3 and TM10 is often referred to as the "crossover" region.

The statement on p. 6 has been modified to “… to form a crossover region with an internal cavity within the membrane”. The other remaining “internal cavity” terms (on p. 13 and p. 23) have been replaced by “crossover region”.

The Abstract is misleading by stating "To elucidate …. in its native form, human ghost membrane was applied to a cryo-EM holey carbon grid to define its composition". While it is true that the band 3 protein is from a native source, it is no longer in its native form after detergent extraction and nor was the ghost membrane directly applied to the grid. When I first read the Abstract, I was expecting that authors would report associated proteins to band 3 or tightly bound native lipids and so on.

The statement in the “Abstract” has been rephrased to “To elucidate the structural mechanism of the band 3 transporter, detergent solubilized human ghost membrane reconstituted in nanodiscs was applied to a cryo-EM holey carbon grid to define its composition” in this revised version of the manuscript.

The word "concerted" is prone to misunderstanding. I understand that the authors use the word to describe a process in which a bound anion is knocked off by an incoming anion, but I think "obligatorily coupled" might work.

The word “concerted” has been changed to “obligatorily coupled”, following this reviewer’s suggestion.


Reviewer #4: In recent years there have been a flurry of papers describing the structure of the dimeric human Band 3 membrane protein using single particle cryo-electron microscopy. Two of these papers [Refs. 22 & 39] describe the ankyrin complex that includes the structure of the entire Band 3 protein interacting with Protein 4.2 and the membrane cytoskeleton (Vallese et al. (2022) <https://pubmed.ncbi.nlm.nih.gov/35835865/>; Xia et al. (2022) <https://www.nature.com/articles/s41594-022-00779-7>).  An important consideration in these studies is that the cytoplasmic domain of purified Band 3 is unstructured in the absence of an interaction with Band 4.2.  Another paper [Ref. 18] describes the structure of the membrane domain of Band 3 with a series of bound inhibitors (Capper, et al. (2023) <https://pubmed.ncbi.nlm.nih.gov/37679563/>).  An additional paper [Ref 23] describes the structure of the membrane domain bovine Band 3 at resolutions of 4.5-7.1 Å in support of an elevator
mode of transport (Zhekova et al. (2022) <https://www.nature.com/articles/s42003-022-04306-8>).  This paper describes Band 3 in the inward facing conformation in addition to the outward facing conformation.  The paper also describes a Band 3 dimer in which the subunits are in the outward and inward-facing conformation.  The first description of the crystal structure of the isolated membrane domain of human Band 3 in the outward-facing state with bound inhibitor was published in 2015 (Arakawa et al. (2015) <https://www.science.org/doi/10.1126/science.aaa4335>).

So, what is new in this paper?

1.      The protein is extracted from membranes with detergent and reconstituted into nanodiscs that retain endogenous lipids that were revealed in the structure, included PIP2 molecules at the dimer interface as predicted from MD simulation studies.
2.      The paper provides the structure of the human Band 3 in five different conformational states at a resolution of Å, including an asymmetrical dimer in both the outward (OF) and inward-facing (IF) states.
3.      The OF/IF structure (#1) at 2.99 Å resolution revealed the positions of chloride close to Asp681 in the outward-facing state while the inward-facing state was devoid of substrates.
4.      The IF/IF state (#2) at 2.97Å resolution was also devoid of substrates, corresponding to the empty inward-facing state.
5.      IF/IF (#3), OF/IF (#4) and OF/OF (#5) structures were obtained in the presence of bicarbonate. IF/IF (#3) at 2.99 Å resolution was also empty like #2.  The OF/IF (#4) state at 3.12 Å resolution had bound bicarbonate interacting with Arg730 as expected, while the IF subunit was once again empty.  The OF/OF (#5) state at 3.16 Å resolution contained bicarbonate in both subunits.
6.      The author note a change in the conformation of TM10 assuming a random state in the inward-facing conformation moving Arg730 ~14 Å towards the inside of the cell.
7.      The authors conclude that the structures support an elevator mode of transport.

This paper provides five highest resolution structures of human Band 3 in various conformational states, including an asymmetrically OF/IF form obtained to date.  The empty and OF state with bound chloride and bicarbonate is provided as is the empty IF state.  The binding sites for chloride and bicarbonate are described in exquisite molecular detail.  It may be difficult to capture the IF state with bound chloride or bicarbonate due to the lower affinity for these ions and accessibility to solvent.

We thank this reviewer’s detailed review and very much appreciate this reviewer’s comment that our findings provide new information to the field.

Minor Comments

1.       I would include the word "human" in the title and in the abstract as appropriate.

The word “human” has been included in the title, following this reviewer’s suggestion.

2.      Only the membrane domain was resolved in these structures and this should be made clear in the abstract and early in the paper.

Yes, we have made clear that only the membrane domain was resolved in this revised manuscript.

3.      I would recommend adding a reference in the Introduction to the excellent review "Cell physiology and molecular mechanism of anion transport by erythrocyte band3/AE1" by Michael Jennings (<https://journals.physiology.org/doi/full/10.1152/ajpcell.00275.2021>).

We thank this reviewer’s suggestion. This is a great reference and has been added in this revised manuscript.

4.      The inward-facing state of Band 3 was also observed in the structure of bovine Band 3.

A statement “…, although the inward-facing structure of bovine band 3 has been reported recently” has been added at the last paragraph of “Introduction” (p. 5) of this revised version of the manuscript.

5.      It would be useful to compare the conformational changes noted in this paper to those of bovine Band 3, beyond rigid body movements of the core domain.

A new paragraph “Recently, a cryo-EM structure of the bovine band 3 dimer, which also includes OF and IF subunits, has been determined. It appears that our human band 3 structure is in good agreement with that of bovine band 3. Superimposition of the structure of the OF subunit of human band 3 to that of bovine band 3 gives rise to an r.m.s.d. of 1.07 Å (for 422 Cα atoms). Likewise, superimposition of the structure of the IF subunit of human band 3 to that of bovine band 3 provides an r.m.s.d. of 1.28 Å (for 422 Cα atoms) (Figure S4)” has been added on p. 8-9 of this revised manuscript to compare the conformations of bovine band 3 and our human band 3 structures.

6.      A brief discussion of why no substrate-bound IF state was observed would be welcome.

A few statements “Because of the closing of the internal cavity and dissembling of ion binding sites at the inward-facing conformational state, the binding of these ions at this state may be more transient and lower in affinity. Based on the structural information, the ions are possibly more accessible to solvent at this state. Therefore, it may be difficult to capture the bound Cl^-^ or HCO_3_^-^ at the inward-facing state” have been added in the “Discussion” section (p. 14) of this revised version of the manuscript to discuss the possibility as to why no substrate-bound IF state was observed.
